# Being Your Cat: What’s Really Going on in Your Feline’s Mind

**DOI:** 10.1017/awf.2023.31

**Published:** 2023-05-05

**Authors:** Sarah Ellis

**Affiliations:** Head of Cat Mental Wellbeing and Behaviour, International Cat Care, UK

This book arrives at a time when cat texts – on their behaviour, what their body language looks like and even how to play with them – are blossoming. As research on cat behaviour, cognition, welfare and their relationship with people is advancing at speed, the cat-related publication boom comes as no surprise, and a book that translates such science into an easily digestible read, as Celia Haddon and Prof. Daniel Mills do, is always a welcome addition. The authorship combination of best-selling author of popular entertaining cat books (Haddon) and scholarly professor (in veterinary medicine; Mills) brings differing angles to the same topic, creating a combined writing style that is easy to read but underpinned with science. For example, you will find anecdotes of Celia’s life including living with pet mice as a child but also an introduction to the early philosophers’ views of emotionality in animals, including cats.

Perhaps the most compelling aspect of the book is the viewpoint from which it is written. Throughout the book “you” does indeed refer to the reader, but not the reader as their self but as if they were a cat. By putting the reader into the mind and body of a cat, the experience of understanding why a cat thinks and behaves the way it does is greatly enhanced.

The Introduction primes this situation by introducing the *umwelt*, and specifically, the cat’s *umwelt* – the world as experienced by the cat – its self world. This sets the scene for the following five chapters where you are taken through what it feels like to exist in a feline body, from kitten to adult and how your senses differ from that of humans.

Chapters 6 focuses on communication through sound, body language, and touch while Chapter 7 is dedicated entirely to scent communication. A full understanding of chemical communication, including both odours and pheromones, is one I find learners can sometimes struggle with, perhaps because it differs so much from our own human-centred communication. However, keeping the reader in the cat’s *umwelt* and introducing some humorous analogies help, for example, considering ‘Nosebook’ for cats (a play on Facebook) and ‘peemail’ (a play on email) are clever and entertaining ways to help learners remember the functionality of chemicals they cannot naturally detect.

Chapters 8 and 9 focus on emotion and cognition – building upon the sensory processing to build up a picture of how the cat sees and feels about the world. In some parts, the science is presented in a manner where you are introduced to the researcher and some of their background (e.g. Jaak Panksepp) while in others, the science remains more anonymous, and I found myself looking through the endnotes at the back of the book to familiarise myself with the relevant references. This created a bit of a to-ing and fro-ing and I think I would have preferred the references for each chapter to have been more easily accessible at the end of each chapter.

Chapter 10 takes a different turn and suddenly you have to think of yourself as a rescue cat and while the information within the chapter was captivating and important, I didn’t feel it flowed on naturally from Chapter 9. At this point the book starts to feel a little more informative with practical information (rather than theoretical). Statistics are provided, expert voices are quoted and references are more heavily peppered with reports, websites and personal communications. It feels that a natural section break could have been useful at this point in the book.

Chapter 11 follows suit with what it would be like to be a pet cat and I swithered in my mind which would have been best to come first – pet or rescue, particularly since the title of the book is *Being Your Cat.* The final chapter focuses on key components to a cat’s welfare – choice and control. This felt a nice introduction to what a cat needs to live a life worth living and references lead the reader to some practical tools to help put this in place.

The book concludes with a couple of Appendices, the first being a list of useful websites and the second a series of answers to “why do cats…” type questions. I think many will find these interesting since they arm the reader with the answers to questions most people ask when they find out you work with cats for a living!

Whether you are a cat fanatic, involved in the academic study of cats or work with them professionally or voluntarily, the science presented in this book marries very well with the easy-reading style of writing, making a pleasantly digestible book that works well both as a precursor to some of the science-heavy texts, or simply as a stand-alone interesting read for all cat lovers.

